# Historical trends and new surveillance of *Plasmodium falciparum* drug resistance markers in Angola

**DOI:** 10.1186/s12936-021-03713-2

**Published:** 2021-04-07

**Authors:** Emily R. Ebel, Fátima Reis, Dmitri A. Petrov, Sandra Beleza

**Affiliations:** 1 Department of Biology, Stanford University, Stanford, CA 94305 USA; 2 Present Address: Department of Pediatrics, Infectious Disease, Stanford University School of Medicine, Stanford, CA 94305 USA; 3 Hospital Regional de Cabinda, C5QW+XP Cabinda, Angola; 4 Department of Genetics and Genome Biology, University of Leicester, Leicester, LE1 7RH UK

**Keywords:** *Plasmodium falciparum*, Angola, Chloroquine, Lumefantrine, Drug resistance, Selection

## Abstract

**Background:**

*Plasmodium falciparum* resistance to chloroquine (CQ) and sulfadoxine-pyrimethamine (SP) has historically posed a major threat to malaria control throughout the world. The country of Angola officially replaced CQ with artemisinin-based combination therapy (ACT) as a first-line treatment in 2006, but malaria cases and deaths have recently been rising. Many classic resistance mutations are relevant for the efficacy of currently available drugs, making it important to continue monitoring their frequency in Angola.

**Methods:**

*Plasmodium falciparum* DNA was sampled from the blood of 50 hospital patients in Cabinda, Angola from October-December of 2018. Each infection was genotyped for 13 alleles in the genes *crt*, *mdr1*, *dhps*, *dhfr*, and *kelch13,* which are collectively involved in resistance to six common anti-malarials. To compare frequency patterns over time, *P. falciparum* genotype data were also collated from studies published from across Angola in the last two decades.

**Results:**

The two most important alleles for CQ resistance, *crt* 76T and *mdr1* 86Y, were found at respective frequencies of 71.4% and 6.5%. Historical data suggest that *mdr1* N86 has been steadily replacing 86Y throughout Angola in the last decade, while the frequency of *crt* 76T has been more variable across studies. Over a third of new samples from Cabinda were ‘quintuple mutants’ for SP resistance in *dhfr*/*dhps*, with a sixth mutation at *dhps* A581G present at 9.6% frequency. The markers *dhfr* 51I, *dhfr* 108N, and *dhps* 437G have been nearly fixed in Angola since the early 2000s, whereas *dhfr* 59R may have risen to high frequency more recently. Finally, no non-synonymous polymorphisms were detected in *kelch13*, which is involved in artemisinin resistance in Southeast Asia.

**Conclusions:**

Genetic markers of *P. falciparum* resistance to CQ are likely declining in frequency in Angola, consistent with the official discontinuation of CQ in 2006. The high frequency of multiple genetic markers of SP resistance is consistent with the continued public and private use of SP. In the future, more complete haplotype data from *mdr1*, *dhfr*, and *dhps* will be critical for understanding the changing efficacy of multiple anti-malarial drugs. These data can be used to support effective drug policy decisions in Angola.

**Supplementary Information:**

The online version contains supplementary material available at 10.1186/s12936-021-03713-2.

## Background

Anti-malarial drugs have long been important tools for malaria control [1]. However, their efficacy is constantly threatened by the evolution of drug resistance in *Plasmodium falciparum* [2]. Multiple *P. falciparum* genes are involved in drug resistance, and selection of them varies by allele, genetic background, and drug environment [3–5]. Therefore, frequent monitoring of resistance alleles is crucial to predicting the spread of drug resistance. This is especially true in the West African country of Angola, where malaria cases and deaths are on the rise [6].

The first anti-malarial drug to enjoy widespread use in Angola was chloroquine (CQ) in the 1950s [7]. CQ resistance was first confirmed in Angola in the 1980s, and by the early 2000s, CQ failure rates exceeded 80% [8, 9]. As a result, CQ was discontinued in Angola in favour of artemisinin-based combination therapy (ACT) starting in 2006 [6]. To discourage the evolution of artemisinin resistance, artemisinin is used in combination with the longer-acting partner drugs lumefantrine or amodiaquine, which is chemically related to CQ [10]. Artemisinin resistance has not yet appeared in Angola, although many partially resistant *kelch13* mutations have emerged in Southeast Asia [5, 11]. Nonetheless, occasional ACT failures have been reported in Angola that could be due to partner drug resistance [6, 12].

Strong *P. falciparum* resistance to CQ and amodiaquine is caused by *crt* 76T, a lysine to threonine substitution at codon 76 of the chloroquine resistance transporter (Table 1). A meta-analysis found this allele to be 7.2-fold overrepresented in CQ treatment failures [13], reflecting its selection by CQ and amodiaquine in many clinical studies (Table 1). In Angola, 76T is typically found on the haplotype *crt* 72–76 CVIET, which is of Asian origin [14]. CQ resistance has also evolved independently through the haplotype *crt* 72–76 SVMNT in South America and Papua New Guinea [15].

**Table 1:**
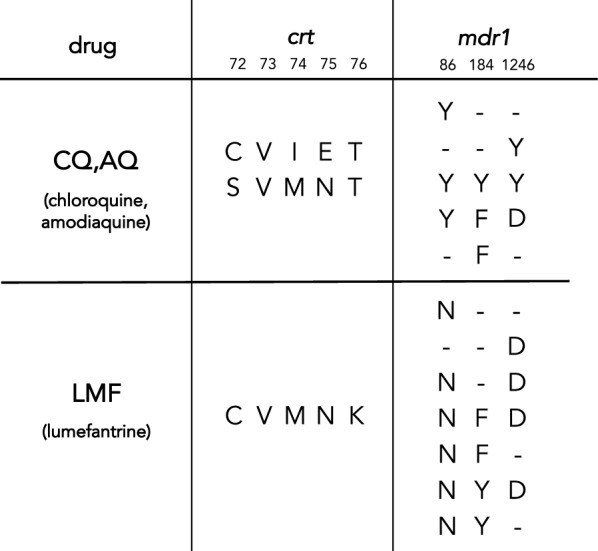
Alleles of *P. falciparum* genes *crt* and *mdr1* that are selected in the presence of chloroquine amodiaquine and lumefantrine

Numbers in the header indicate amino acid position. Incomplete haplotypes for *mdr1* are shown as reported in the literature, though in most cases the causal alleles are unclear. Additional details and references are available in Additional file 1: Table S1

The 86Y allele of *mdr1*, or multidrug resistance gene 1, also confers resistance to CQ and amodiaquine [13]. Although this specific polymorphism dominated early studies of *mdr1* and CQ resistance, the evolution of *mdr1* is complicated by linkage between position 86 and other functional polymorphisms [16]. Precise *mdr1* haplotypes vary among *P. falciparum* populations and drug settings, but in Angola alone, at least six alleles at three *mdr1* positions have been proposed to modulate resistance to CQ, amodiaquine, and lumefantrine (Table 1, Additional file 1: Table S1).

The drug sulfadoxine-pyrimethamine (SP) has also been in widespread use in many African countries since the 1960s [17]. *Plasmodium falciparum* quickly began evolving partial resistance to SP, mediated by numerous substitutions in *dhps* and *dhfr* [18]. The risk of SP treatment failure increases with the number of mutant alleles present, with “quintuple mutants” at codons 437/540 of *dhps* and codons 51/59/108 of *dhfr* of particular concern [19–21]. By the early 2000s, these alleles were common in Angola and 25–39% of *P. falciparum* infections failed to respond to SP treatment [9]. SP has since been discontinued as a first-line therapy in Angola, but it is still available at private pharmacies [22], where it comprised 10–40% of all anti-malarial sales in Huambo between 2009 and 2013 [23]. Intermittent, preventative SP is also recommended for pregnant women in Angola [24], although data from 2015 to 2016 indicate that only 30–40% of pregnant women actually received it during prenatal visits [25]. In other African countries, additional *dhps* mutations are emerging that appear to threaten the efficacy of SP treatment [26, 27]. It is, therefore, critical to continue monitoring genetic variation in *dhfr* and *dhps*.

In this work, 50 *P. falciparum* infections from Cabinda, Angola were genotyped for 13 markers of drug resistance in the genes *crt*, *mdr1*, *dhps*, *dhfr*, and *kelch13*. Similar data were also gathered from studies published on Angolan *P. falciparum* in the last two decades. For every gene but *kelch13*, the observed temporal patterns of allele frequency change are consistent with the current usage and availability of anti-malarial drugs. This work can inform future decisions on drug administration in Angola.

## Methods

### Sample collection and ethics statement

Patients reporting to the Hospital Regional de Cabinda between October and December 2018 with fever, chills, or other malaria symptoms were offered the option to be consented to this study. Sample collection followed protocols approved by Stanford University (IRB #39149) and the Medical Ethics Committee of the University 11^th^ of November in Cabinda. In all, 8 children under 5 years, 14 children under 18 years, and 28 adults between 18 and 55 years were consented to participate. Participants’ blood was drawn from a vein and screened under a microscope for *P. falciparum* parasites. Parasitaemia was determined for adult participants and ranged from 1,787–35,424 (mean: 13,617) parasites/uL. All positive, whole blood samples were filtered through cellulose columns to remove leukocytes [28], and the filtered red blood cells were spotted on Whatman FTA cards (Sigma Aldrich), dried, and stored for at least 6 months.

### DNA extraction and genotyping

To elute DNA, saturated circles were cut out of the Whatman FTA cards and incubated in 800 uL TE buffer (10 mM Tris–Cl, 1 mM EDTA, pH 8.0) with 20 uL Proteinase K (Invitrogen) for 2 h at 65 °C. DNA was extracted from the liquid supernatant using a phenol–chloroform protocol with phase-lock gel tubes [29].

PCR amplification of the *P. falciparum* genes *crt, mdr1, dhfr, dphs,* and *kelch13* was performed with previously published primers [30–32]. Cycling protocols were based on manufacturer recommendations for OneTaq Hot Start 2X Master Mix (NEB) and/or Phusion High-Fidelity PCR Master Mix with HF Buffer (NEB) (Additional file 2: Table S2). Reactions were visualized in 1% agarose gels, and if successful, cleaned with ExoSAP-IT (ThermoFisher) and Sanger sequenced (Elim Bio). Sanger chromatogram data were compared to PlasmoDB reference *P. falciparum* sequences using Benchling. Analysis was focused on amino acid substitutions in the following positions: *mdr1* N86Y, Y184F, and D1246Y; *crt* CMNVK 72–76 CVIET; *dhfr* C50G, N51I, C59R, and S108N; *dhps* S436A, A437G, K540E, and A581E. For *kelch13*, the entire amplified fragment spanning codons 389–649 was examined for polymorphisms.

### Mixed infections and allele frequency calculations

For each sample, a mixed infection was inferred if the sequencing chromatogram showed equally sized, double peaks for any of the 13 analysed loci. The frequency of each allele was determined based on the total number of infections, with single infections contributing one genotype and mixed infections contributing two. These calculations are available in Additional file 5: Table S5. The 43 samples without missing data at *dhfr* or *dhps* were also assessed for the presence of seven SP-resistance alleles (*dhfr-*51I, *dhfr-*59R, *dhfr-*108N, *dhps*-436A, *dhps*-437G, *dhps*-540E, *dhps*-581G) [33, 34].

### Collection of historical data

Publications reporting the prevalence of drug-resistance loci anywhere in Angola since 1995 were gathered from a recent review [7] and from the Worldwide Antimalarial Resistance Network (WWARN) Molecular Surveyor tool (http://www.wwarn.org/molecularsurveyor/). To avoid biases imposed by drug selection, studies were excluded if they selected samples for genotyping based on later treatment failure. In cases where details on mixed infections were provided, the original data were used to calculate allele frequencies as described above; other cases were treated as having 0 mixed infections. These calculations are available in Additional file 5: Table S5.

## Results

### Genotyping success and mixed infections

Each sample was successfully genotyped at an average of 12 out of 13 loci (Additional file 3: Table S3). The *kelch13* locus had the highest success rate (100%), while *crt* had the lowest success rate (78%). The *crt* primers used here have performed well on other Angolan samples [30], but in this cohort, even the nested protocol amplified products of multiple sizes (Additional file 6: Fig. S1). Fifteen of 50 samples had sequence diversity (i.e., peaks of two bases) in at least one resistance marker site, indicating the presence of at least two genetically distinct strains.

### Very little polymorphism in *kelch13*

Across the amplified *kelch13* fragment, which spanned codons 389–649, only one synonymous polymorphism (R471R) was observed in one sample. No *kelch13* polymorphisms were observed at any sequenced codons that have been linked to delayed parasite clearance in the presence of artemisinin by a recent meta-analysis by the Worldwide Antimalarial Resistance Network (WWARN) [35].

### Markers of CQ resistance in *crt *and *mdr1*

The *crt* 72–76 CVIET haplotype, which confers strong resistance to CQ, was detected in 73% of single isolates in this study (Table 2). Using a definition of allele frequency that incorporates mixed isolates (Methods; Additional file 5: Table S5), 76T was estimated to occur at 70.7% frequency in Cabinda in 2018 (Table 3). In contrast, earlier data from four other Angolan provinces collected between 2004 and 2011 indicated 76T at 89–97% frequency (Table 3). Later studies of Chinese migrant workers, sampled from unspecified locations within Angola in 2013 and 2014, estimated the frequency of 76T to be between 30 and 44%. Overall, despite appreciable variation across studies and sites, *crt* 76T appeared to be rarer in Angola in the 2010s than it was in the 2000s (Table 3).

**Table 2:** Prevalence of *P. falciparum* drug resistance marker haplotypes in Cabinda, Angola in 2018

	N	Prevalence
*crt* 72–76		
CVIET	27	73.0%
CVMNK	10	27.0%
Mixed	2	–
Missing	11	–
*mdr1* 86-184-1246		
NYD	21	55.3%
NFD	14	36.8%
YFD	1	2.6%
YYD	1	2.6%
YYY	1	2.6%
Mixed	4	–
Missing	8	–
*dhfr/dhps* 51-59-108/436-437-540–581		
** IRN**S**G**KA (4)	13	40.6%
** IRN**S**GE**A (5)	12	37.5%
** IRNAG**KA (5)	5	15.6%
** I**C**NAG**KA (4)	1	3.1%
** IRNAG**K**G** (6)	1	3.1%
Mixed	11	–
Missing	7	–

Only single infections with complete haplotype data contribute to the prevalence estimates shown here. Additional data for mixed infections and partial haplotypes are available in Additional file 3: Table S3. For *dhfr/dhps*, amino acids associated with SP resistance [33, 34] are indicated in bold text with the total number of resistance alleles per haplotype in parentheses. See Table 1 for how *crt* and *mdr1* markers correspond to sensitivity to different drugs

**Table 3:** Frequency of the CQ-resistant *crt* 76T allele in Angola since the early 2000s

Year	76T	Province	Recruitment	Notes	Ref
2004	93.9%	Uíge	Children 4–108 months in hospital emergency unit	–	[36]
2007	93.9%	Luanda	Children 1–16 years with uncomplicated malaria in hospital	Year approximate	[54]
2007	97.1%	Luanda	Adults > 18 years with uncomplicated malaria	76T primarily on SVMNT haplotype	[30]
2010	50.7%	Bengo	Baseline prevalence survey in women and children	–	[55]
2010–11	88.9%	Benguela	Random household survey of children < 15 years	Mixed infections not mentioned	[56]
2012–14	44.4%	–	Migrant workers returning to China	–	[57]
2012–15	29.9%	–	Migrant workers returning to China	–	[58]
2012–16	38.1%	–	Migrant workers returning to China	Mixed infections not mentioned	[59]
2018	70.7%	Cabinda	Adults and children in hospital	–	This study

Each allele frequency was re-calculated from published prevalence data to include mixed infections whenever possible (see Additional file 5: Table S5)

The *mdr1* allele 86Y, which also confers resistance to CQ, was detected in just 7.8% of single infections in Cabinda in 2018 (Table 2) and had an allele frequency of 6.5% when also considering mixed infections (Table 4). Accordingly, the alternate allele N86—which is associated with both CQ-sensitivity and artemether-lumefantrine (AL) treatment failure (Table 1)—had an allele frequency of 93.5% (Table 4). The linked polymorphism *mdr1* 184F was present at 44.0% frequency (Table 4). These estimates are substantially higher than those from all other studies conducted in Angola in the last 15 years (Table 4), which together suggest an increase of N86 and perhaps 184F across multiple sites over time. A single isolate from Cabinda in 2018 also contained the additional CQ-resistance allele *mdr1* 1246Y (Table 1), which occurred on an 86Y/Y184 background (Table 2).

**Table 4:** Frequencies of *P. falciparum* drug resistance markers in *mdr1* in Angola since the early 2000s

Year	86Y	184F	Province	Recruitment	Notes	Ref
2003	73.3%	–	Luanda (+ São Tomé)	Travellers returning to Portugal	Year approximate; mixed infections reported as dominant allele	[60]
2004	51.7%	–	Uíge	Children 4–108 months in hospital emergency unit	-	[36]
2007	64.8%	–	Luanda	Children 1–16 years with uncomplicated malaria in hospital	Year approximate	[54]
2007	65.5%	17.9%	Luanda	Adults > 18 years with uncomplicated malaria	Frequent failures to amplify *mdr1*	[30]
2010	27.8%	34.8%	Bengo	Baseline prevalence survey in women and children	-	[55]
2010–2011	53.7%	14.8%	Benguela	Random household survey of children < 15 years	Mixed infections not mentioned	[56]
2010–2013	24.5%	–	Luanda	Adults and children > 6 months with uncomplicated malaria in health care units	-	[62]
2012–2016	14.4%	–	–	Migrant workers returning to China	Mixed infections not mentioned	[59]
2015	15.3%	31.5%	Benguela, Lunda Sul, Zaire	Children 6–108 months with uncomplicated malaria	Mixed infections reported as mutant	[62]
2018	6.5%	44.0%	Cabinda	Adults and children in hospital	-	This study

Each allele frequency was re-calculated from published prevalence data to include mixed infections whenever possible (see Additional file 5: Table S5)

### Multiple markers of SP resistance are common in Angola

Over one third (37.5%) of *P. falciparum* isolates sampled here were “quintuple mutants” for five *dhfr* and *dhps* alleles that confer strong SP resistance (**IRN**S**GE**A, Table 2). For comparison, this haplotype was not observed among 66 samples collected in Uíge in 2004 [36], and was present in only 11.6% of samples from migrant workers collected between 2013–2016 [37]. A different set of five markers (**IRNAG**KA) was found in 15.6% of isolates in this study, while 43.7% contained four markers (**IRN**S**G**KA or **I**C**NAG**KA). No infection contained three or fewer markers (Table 2). One single infection contained six markers (**IRNAG**K**G**), as did two mixed infections (Additional file 3: Table S3); however, the 436A allele present on these haplotypes is considered less important for SP resistance than 540E [34], which was observed only in the canonical “quintuple” mutant (Table 2).

It was not straightforward to compare the prevalence of *dhfr* and *dhps* haplotypes across studies, due to inconsistencies in which amino acids were reported; how missing data were treated; and the relatively common practice of reporting *dhfr* and *dhps* haplotypes separately (Table 5; Additional file 4: Table S4). With these caveats, however, it was possible to examine the frequencies of individual amino acid variants in Angola over time (Table 5). Almost every study conducted between 2004 and 2018 found the mutant alleles *dhfr* 51I, *dhfr* 108N, and *dhps* 437G at high frequencies (85–99%, Table 5), consistent with constant selective pressure from continued use of SP. The mutant alleles *dhfr* 59R, *dhps* 581G, and especially *dhps* 540E were also detected at their highest frequencies within the most recent sample from Cabinda. However, only the data for *dhfr* 59R were consistent with the steady rise of the mutant allele over time in multiple parts of the country (Table 5).

**Table 5:** Frequencies of seven SP-resistance markers in *dhfr* and *dhps* in Angola since the early 2000s

Year	*dhfr*			*dhps*				Province	Recruitment	Notes	Ref
	51I	59R	108N	436A	437G	540E	581G				
2004	–	–	–	56.4	94.9	0	-	Uíge	Children < 12 years at hospital with uncomplicated malaria	Mixed infections reported as mutant or excluded	[63]
2004	95.5	36.4	97	51.5	92.4	0	0	Uíge	Children 4–108 months in hospital emergency unit	Mixed infections reported as mutant	[36]
2007	–	33.1	–	–	–	11.2	–	Luanda	Children 1–16 years with uncomplicated malaria in hospital	Year approximate	[54]
2007	90.9	38	99.1	–	84.8	12.2	–	Huambo, Uíge, Kwanza Norte, Cabinda, Malanje	Baseline survey of children < 5 years	-	[50]
2007	93.4	57.4	98.4	23.3	93.3	10	6.7	Luanda	Adults with uncomplicated malaria at health facilities	*dhfr/dhps* reported separately	[32]
2011	46.2	63.5	98.1	3.4	96.6	6.9	0	Huíla	> 12 years old with uncomplicated malaria in hospital	Mixed infections reported as mutant; *dhfr/dhps* reported separately	[64]
2010–2011	89.7	25.9	96.6	26.7	86.7	0	0	Benguela	Random household survey of children < 15 years	Mixed infections not mentioned; *dhfr/dhps* reported separately	[56]
2013–2016	94.2	73.9	98.6	4.3	91.3	17.4	0	–	Migrant workers returning to China	Mixed infections reported as mutant	[37]
2018	97.9	93.8	94	23.5	97.9	30.6	9.6	Cabinda	Adults and children in hospital	-	This study

Each allele frequency was re-calculated from published prevalence data to include mixed infections whenever possible (see Additional file 5: Table S5)

## Discussion

Discontinuation of CQ in several African countries including Malawi, the Gambia, Kenya, Ethiopia, Tanzania, and Grand Comore has led to major declines of *crt* 76T [38–43], the most important allele for CQ resistance in *P. falciparum*. Six studies conducted in Angola since 2010 similarly found reduced frequencies of *crt* 76T compared to the early 2000s (Table 3). However, precise frequency estimates have ranged widely among studies (30–89%, Table 3), perhaps because of local differences in anti-malarial use or parasite diversity. CQ is still available in some Angolan pharmacies, although it comprised fewer than 1% of sales in Huambo between 2009 and 2013 [23]. Another important source of selection could be the ACT partner drug amodiaquine (Table 1), which is found in one of two artemisinin-based combinations currently implemented in Angola. More information on anti-malarial drug usage across the country, as well as continued genetic monitoring of *P. falciparum*, will be useful for understanding the evolution of *crt* in Angola.

Discontinuation of CQ in Grand Comore and the Gambia also preceded the decline of *mdr1* 86Y [38, 40], the second most important allele for CQ resistance in *P. falciparum*. Several studies from across Angola are consistent with a steady decline of 86Y after 2007, with the exception of a 2011 household survey in Benguela (Table 4). One possible explanation for the faster loss of *mdr1* 86Y, as opposed to *crt* 76T, could be that *mdr1* N86 or its linked alleles are involved in low-level lumefantrine resistance (Table 1; Table S1). Lumefantrine is a component of artemether-lumefantrine (AL), the most common ACT currently implemented in Angola [6, 44]. Since 2013, three large studies have reported that AL treatment efficacy in Zaire or Lunda Sul fell below the WHO standard of 90% [12, 45, 46]. These treatment failures have been interpreted as signs of decreased susceptibility to lumefantrine in the parasite population [12], although the genetic basis of lumefantrine resistance has yet to be conclusively demonstrated and could involve multiple loci. Further studies on the mechanisms of lumefantrine tolerance in *P. falciparum* will be key to enabling molecular monitoring in the future.

In contrast to markers of CQ resistance, markers of SP resistance have been on the rise in several African countries [40, 41, 47–49] where SP is used for malaria treatment and prevention. In Angola, the available data suggest a rapid increase in the prevalence of SP-resistant “quintuple mutants” in *dhfr/dhps* between 2004 (0%) [36], 2013–2016 (11.6%) [37], and 2018 (Table 2, 37.5%). This increase could be partially driven by the rise of one particular marker, *dhfr* 59R (Table 5), although *dhfr/dhps* genetic diversity may also vary among sites [50]. In Angola, it is likely that unregulated consumer use of SP [22, 23] and use of SP for intermittent preventive treatment in pregnancy [51] are sources of selection for SP resistance. This situation should continue to be monitored closely to avoid the eventual loss of important SP benefits during pregnancy [27, 52]. In the future, complete reporting of haplotype information for all combined *dhfr/dhps* alleles in each *P. falciparum* infection (e.g. Additional file 3: Table S3) will help accomplish this goal.

These interpretations of allele frequency change in Angola over time are subject to a number of limitations. First, the historical data were drawn from studies conducted in several geographical locations across Angola (Tables 3, 4, 5; Additional file 4: Table S4). Because provinces vary in patterns of malaria transmission [6] and anti-malarial availability [22, 23], selection on resistance alleles is not expected to be uniform across the entire country. Second, each study varied in its sampling and many also varied in their reporting of mixed infections (Tables 3, 4, 5; Additional file 4: Table S4), which has the potential to reduce or inflate estimates of mutant allele frequencies. Third, several studies (including this one) provide data for a relatively limited number of subjects, which may also lead to biases in allele frequency estimates. Despite these limitations, however, the apparent trends of drug resistance alleles over time (Tables 3, 4, 5) are consistent with the common usage of ACT and SP and discontinuation of CQ across Angola since the mid-2000s.

Finally, we detected no signs of alleles in *kelch13* that confer partial resistance to artemisinin. This result is consistent with the lack of evidence in Africa for the spread of alleles that diminish ACT efficacy, although some countries have recently reported the presence of validated markers [53]. Continued monitoring of *kelch13* in Africa is important, as artemisinin remains the cornerstone of anti-malarial drug policy in many countries.

## Conclusions

Multiple drug resistance alleles in Angolan *P. falciparum* have experienced changes in frequency since CQ was officially discontinued in 2006. Markers of resistance to CQ appear to be declining, but markers of strong SP resistance and potential low-level lumefantrine resistance are rising. Continued monitoring and drug policy adjustments will be necessary in the future to regain control of *P. falciparum* malaria in Angola.

## Supplementary Information

**Additional file 1**: **Table S1**. Evidence and references supporting summary in Table 1 (XLSX 11 KB)

**Additional file 2**: **Table S2**. PCR primers and cycling conditions (XLSX 14 KB)

**Additional file 3**: **Table S3**. Full genotype results from Cabinda for the 13 loci (XLSX 13 KB)

**Additional file 4**: **Table S4**. Summary of 17 studies of P. falciparum drug resistance markers in Angola (XLSX 13 KB)

**Additional file 5**: **Table S5**. Calculations of allele frequencies from published count data. Mixed infections, if reported, were considered to contribute two genotypes. If not reported, or insufficiently reported, zero mixed infections were assumed. The year indicates date of sampling (XLSX 19 KB)

**Additional file 6**: **Figure S1**. PCR with published crt primers amplified multiple bands. The expected product was <100 bp. In some but not all cases, it was still possible to determine the sequence for amino acids 72-76 using these reactions (DOCX 439 KB)

## Data Availability

The new *P. falciparum* genotype data presented here may be found in Supplementary Table 3.
