# Supplementary material for: Historical trends and new surveillance of Plasmodium falciparum drug resistance markers in Angola
Source: Malar J. 2021 Apr 7;20:175. doi: 10.1186/s12936-021-03713-2 (PMC8028775; doi:10.1186/s12936-021-03713-2)
Supplement: Supplementary file 6 — Additional file 6: Figure S1. PCR with published crt primers amplified multiple bands. The expected product was <100 bp. In some but not all cases, it was still possible to determine the sequence for amino acids 72-76 using these reactions (DOCX 439 KB) [file 12936_2021_3713_MOESM6_ESM.docx]

**Table S1. Evidence and references supporting summary in Table 1.**

1. Djimdé, A. *et al.* A Molecular Marker for Chloroquine-Resistant Falciparum Malaria. *N. Engl. J. Med.* **344**, 257–263 (2001).

2. Picot, S. *et al.* A systematic review and meta-analysis of evidence for correlation between molecular markers of parasite resistance and treatment outcome in falciparum malaria. *Malar. J.* **8**, 89 (2009).

3. Holmgren, G. *et al.* Amodiaquine resistant Plasmodium falciparum malaria in vivo is associated with selection of pfcrt 76T and pfmdr1 86Y. *Infect. Genet. Evol.* **6**, 309–314 (2006).

4. Ursing, J., Kofoed, P.-E., Rodrigues, A., Rombo, L. & Gil, J. P. *PLASMODIUM FALCIPARUM GENOTYPES ASSOCIATED WITH CHLOROQUINE AND AMODIAQUINE RESISTANCE IN GUINEA-BISSAU*. (2007).

5. Mandi, G., Mockenhaupt, F. P., Coulibaly, B., Meissner, P. & Müller, O. Malaria Journal Efficacy of amodiaquine in the treatment of uncomplicated falciparum malaria in young children of rural north-western Burkina Faso. (2008) doi:10.1186/1475-2875-7-58.

6. Wootton, J. C. *et al.* Genetic diversity and chloroquine selective sweeps in Plasmodium falciparum. *Nature* **418**, 320–323 (2002).

7. Somé, A. F. *et al.* Selection of known plasmodium falciparum resistance-mediating polymorphisms by artemether-lumefantrine and amodiaquine-sulfadoxine-pyrimethamine but not dihydroartemisinin-piperaquine in Burkina Faso. *Antimicrob. Agents Chemother.* **54**, 1949–1954 (2010).

8. Sisowath, C. *et al.* In vivo selection of Plasmodium falciparum parasites carrying the chloroquine-susceptible pfcrt K76 allele after treatment with artemether-lumefantrine in Africa. *J. Infect. Dis.* **199**, 750–757 (2009).

9. Danquah, I. *et al.* Selection of pfmdr1 and pfcrt alleles in amodiaquine treatment failure in north-western Burkina Faso. *Acta Trop.* **114**, 63–66 (2010).

10. Humphreys, G. S. *et al.* Amodiaquine and artemether-lumefantrine select distinct alleles of the Plasmodium falciparum mdr1 gene in Tanzanian children treated for uncomplicated malaria. *Antimicrob. Agents Chemother.* **51**, 991–997 (2007).

11. Okell, L. C. *et al.* Emerging implications of policies on malaria treatment: Genetic changes in the Pfmdr-1 gene affecting susceptibility to artemether–lumefantrine and artesunate–amodiaquine in Africa. *BMJ Glob. Heal.* **3**, 999 (2018).

12. Happi, C. T. *et al.* Selection of Plasmodium falciparum multidrug resistance gene 1 alleles in asexual stages and gametocytes by artemether-lumefantrine in nigerian children with uncomplicated falciparum malaria. *Antimicrob. Agents Chemother.* **53**, 888–895 (2009).

13. Nzila, A., Okombo, J., Ohuma, E. & Al-Thukair, A. Update on the in vivo tolerance and in vitro reduced susceptibility to the antimalarial lumefantrine. *J Antimicrob Chemother* **67**, 2309–2315 (2012).

14. Lobo, E. *et al.* Prevalence of pfmdr1 alleles associated with artemether-lumefantrine tolerance/resistance in Maputo before and after the implementation of artemisinin-based combination therapy. *Malar. J.* **13**, 300 (2014).

15. Mungthin, M. *et al.* Association between the pfmdr1 gene and in Vitro artemether and lumefantrine sensitivity in thai isolates of Plasmodium falciparum. *Am. J. Trop. Med. Hyg.* **83**, 1005–1009 (2010).

16. Foote, S. J. *et al.* Several alleles of the multidrug-resistance gene are closely linked to chloroquine resistance in Plasmodium falciparum. *Nature* **345**, 255–258 (1990).

17. Thomsen, T. T. *et al.* Rapid selection of Plasmodium falciparum chloroquine resistance transporter gene and multidrug resistance gene-1 haplotypes associated with past chloroquine and present artemether-lumefantrine use in Inhambane District, Southern Mozambique. *Am. J. Trop. Med. Hyg.* **88**, 536–541 (2013).

18. Ljolje, D. *et al*. Prevalence of molecular markers of artemisinin and lumefantrine resistance among patients with uncomplicated *Plasmodium falciaprum* malaria in three provinces in Angola, 2015. *Malar. J.* **17**, 84 (2018).

19. Venkatesan, M. *et al*. Polymorphisms in *Plasmodium falciparum* Chloroquine Resistance Transporter and Multidrug Resistance 1 Genes: Parasite Risk Factors that Affect Treatment Outcomes for *P. falciparum* Malaria after Artemether-Lumefantrine and Artesunate-Amodiaquine. *Am. J. Trop. Med. Hyg.* **91**, 833–843 (2014).

**Table S2. PCR primers and cycling conditions.**

**Table S3. Full genotype results from Cabinda for the 13 loci.**

**Table S4. Summary of 17 studies of *P. falciparum* drug resistance markers in Angola.**

**Table S5. Calculations of allele frequencies from published count data**. Mixed infections, if reported, were considered to contribute two genotypes. If not reported, or insufficiently reported, zero mixed infections were assumed. The year indicates date of sampling.

**Figure S1. PCR with published *crt* primers amplified multiple bands.** The expected product was <100 bp. In some but not all cases, it was still possible to determine the sequence for amino acids 72-76 using these reactions.
